# Temporal establishment of the colon microbiota in Angus calves from birth to post-weaning

**DOI:** 10.1371/journal.pone.0334261

**Published:** 2025-10-28

**Authors:** Michelle M. Stafford, Paul E. Smith, Sinead M. Waters, Frank Buckley, Steven McLoughlin, Stuart F. Kirwan, Eoin O’Hara, David A. Kenny

**Affiliations:** 1 Teagasc Animal and Bioscience Research Department, Teagasc Grange, Meath, Ireland; 2 School of Biological, Earth and Environmental Sciences, University College Cork, Cork, Ireland; 3 School of Biological and Chemical Sciences, University of Galway, University Rd, Galway, Ireland; 4 School of Biological Sciences, Queens University Belfast, Belfast, United Kingdom; 5 Department of Agricultural, Food, & Nutritional Sciences, University of Alberta, Edmonton, Canada; 6 Agriculture and Agri-Food Canada, Lethbridge Research and Development Centre, Lethbridge, Canada; University of Illinois Urbana-Champaign, UNITED STATES OF AMERICA

## Abstract

During the peri- and early post-partum period, the gastrointestinal tract (GIT) of the calf is colonised by a diverse microbiota. In the colon, this microbial community contributes to digestive activities, immune modulation, and overall calf health and productivity. However, the current knowledge of temporal microbial establishment in the neonatal hindgut is limited. This study focused on the ontogeny of colon microbiota establishment in Aberdeen Angus cross beef calves located across two farms, from birth through to post-weaning. Colon digesta samples were obtained from calves euthanised on days D0 (n = 7), D7 (n = 7), D14 (n = 5), D21 (n = 7), D28 (n = 5), and D96 (n = 7) of life. 16S rRNA amplicon sequencing was used to determine prokaryotic community composition. The alpha (α) and beta (β) diversity were assessed with age and farm included as fixed effects. Bacterial α-diversity increased significantly with age, showing changes in relative abundance between D7 and later stages, including D21, D28, and D96 (each p < 0.0001). PERMANOVA analysis indicated no significant difference in microbial composition between D14 and D21 (P = 0.22), suggesting a period during which compositional changes were reduced, reflecting a temporary phase of relative similarity in the colon microbiota. However, weaning altered the colon microbiota, as evidenced by the differences were observed between D28 and D96 (P < 0.001) suggested compositional shifts associated with weaning, accompanied by increased α-diversity. These observations will help to inform the design of the future, large scale studies aimed at understanding how early life microbial dynamics influence calf health, welfare, and future productive performance.

## Introduction

The gastrointestinal tract (GIT) plays a key role in ruminant livestock production systems where its resident microbiome is vital for immune system establishment and maintenance [1], nutrient supply [2], disease prevention [3], and overall animal health [4]. At birth, the rumen is undeveloped, and microbial fermentation in the lower gut is especially important in the early-life development in ruminants. In particular, the colon microbiome contributes to modulating host defence mechanisms and contributes to the regulation of inflammatory response, both of which are essential for adaptation to the environment and dietary changes during the neonatal period [5,6]. Unlike humans, calves are born almost entirely agammaglobulinemic and do not experience cross-placental transfer of immunoglobulins due to the cotyledonary synepitheliochorial placental structure [7,8]. Instead relying on the passive transfer of colostrum antibodies from the dam for immune protection [9]. Failure of this passive transfer is associated with increased risk of diarrhoea, respiratory diseases and calf [10].

In addition to its role in immune development, the neonatal GIT undergoes a critical period of microbial assembly and succession, during which early colonisers shape the trajectory of the developing microbiota. Microbial community assembly in early life is driven by a combination of stochastic and deterministic processes that influence diversity, stability, and function [11–13]. The establishment of a resilient and functionally robust gut microbiome is important for supporting host health and development, with early perturbations having long-lasting consequences. Understanding the patterns of microbial succession, including phases of rapid change and transient stability, is therefore critical for optimising neonatal management strategies aimed at promoting long-term animal health and performance.

The GIT is a low biomass environment, resulting in the multiple challenges for performing reliable microbial analysis [14]. Molecular methods used to identify bacteria in low-burden environments have limitations, which can lead to results influenced by background noise and false positives [15]. While colonisation of the GIT was previously hypothesised to occur *in utero* [16], the hypothesis has now shifted with the initial colonisation widely accepted to occur promptly following birth [17]. The initial microbial colonisation of the neonatal GIT is postulated to play a crucial role in developing the adaptive immune system of ruminants, promoting colonisation resistance and shaping mucosal immunity [18,19]. Beneficial microbes are also involved in the expression of tight junction protein and B-cell activation, contributing to intestinal homeostasis and barrier function [20].

Microbial establishment and succession of the ruminant GIT during early life is influenced by multiple factors including delivery method, diet, material microbiomes, antibiotic use, colostrum quality and other environmental factors [18,20]. The neonatal lower GIT microbial community is a diverse and dynamic environment. Poor management practises may compromise this community, with microbial dysbiosis adversely affecting host immune function [21]. Gastrointestinal enteritis can be caused by an influx of viral, bacterial or protozoal pathogens [22] and results in increased morbidity and mortality [23]. This can have substantial lasting effects on neonatal calves, resulting in a decrease of the average daily gain (ADG), fertility performance and first lactation milk yield in dairy cattle [24].

Increased access to high-throughput sequencing technology has enhanced the overall understanding of gastrointestinal microbial communities, and their relationship with host health and performance. There is limited information on the temporal colonisation of the neonatal colon, with most prior research focusing on rumen [25], faecal [26], or ileal [19] microbiota. Despite the colon’s central role in immune regulation and fermentation during early life, few studies have examined its microbial succession using direct, destructively sampled colon digesta.

This study aimed to provide preliminary insights into the temporal establishment of the colon microbiota in Aberdeen Angus calves. By examining destructively sampled colon digesta from animals at key early life stages across two farms, we sought to identify age- and environment-associated trends in microbial diversity and composition. We hypothesised that microbial succession in the colon would follow a distinct, age-dependent trajectory from birth through weaning, with marked compositional changes driven by dietary transitions and environmental exposure.

## Materials and methods

### Ethical statement

All experimental procedures described in this paper were approved and conducted with the approval of the Teagasc Animal Ethics Committee (TAEC) and the Irish Health Products Regulatory Association (HPRA; Project Authorisation AE19132/P025).

### Experimental design, animal trial and sample collection

Animal management protocols were previously described in detail elsewhere [25,27]. Briefly, 93 commercially purchased Aberdeen Angus Heifers were housed in Teagasc Mellows Campus, Athenry, Co. Galway, Ireland (Farm 1; F1). The heifers were enrolled in an oestrous synchronisation program and were subsequently artificially inseminated with semen from a single Aberdeen Angus bull sire. This resulted in 67 viable pregnancies, with a subset of 42 heifers utilised for this experiment that were divided into four replicates. This selection was based on logistical and resource constraints, including facility capacity, sampling resources, and the need to balance group sizes across time points. The number of animals used was minimized in accordance with ethical guidelines (principle of Reduction), and the study was conducted to assess the feasibility of longitudinal destructive sampling and to generate preliminary insights into microbial colonisation patterns in the calf. This assured that no more animals than necessary were used to achieve the study’s scientific objectives. The heifers in replicates 1 and 2 (n = 21), calved in their original location F1. The heifers in replicates 3 and 4 (n = 10), were transported to the Department of Agriculture, Food and Marine (DAFM) Longtown Research Facility in Clane, Co. Kildare, Ireland (Farm 2; F2) for calving. Every effort was made to minimise the stress during transport, including careful handling and immediate housing under comparable conditions. The transport was lasted approximately two hours, the pregnant heifers were handled with considerable care, rehoused immediately using comparable conditions to F1, minimising their stress.

Both facilities adhered to the same housing and feeding practices. The heifers were housed indoors for the 8 weeks prior to their expected calving date with *ad libitum* access to moderate energy density grass silage and 2 kg concentrate feed daily. One week prior to their projected date of calving, heifers were assigned to the treatment group which reflected the date on which their calf would be euthanised for sampling, balanced to ensure an even number of male and female calves at each time point. This resulted in the following groups: day 7 (D7; n = 7, F1; n = 5, F2; n = 2), day 14 (D14; n = 5, F1; n = 3, F2; n = 2), day 21 (D21; n = 7, F1; n = 5, F2; n = 2), day 28 (D28; n = 5, F1; n = 4, F2; n = 1), and day 96 (D96; n = 7, F1; n = 4, F2; n = 3). Approximately on the expected calving date, heifers were administered with 2 ml of prostaglandin F2_α_ analogue (Estrumate^TM^, Merck, NJ, USA) to induce parturition.

All calves were delivered trans-vaginally. Calves assigned to D0 had no contact with the cow or their external environment following birth. Calves were euthanised via lethal intravenous injections of pentobarbital sodium. Death was determined by the absence of cardiac activity and the lack of a corneal reflex. No prior anaesthesia or analgesia was administered before euthanasia, as the procedure was performed as a single, rapid, and humane act consistent with AVMA and EU guidelines for euthanasia of livestock. All efforts were made to minimize distress and handling time before the procedure. Euthanasia was scheduled promptly following morning feeding to minimise fasting time and potential discomfort. All procedures were performed by trained personnel to ensure animal welfare was prioritised at all times.

Following euthanasia, the GIT was promptly removed using sterile instruments. Colon digesta was collected by tying off the colon below the cecal junction, the colon digesta was pushed through and stored in a sterile tube. Samples were snap frozen in liquid nitrogen and subsequently stored at −80 °C pending molecular analysis. All other calves suckled their dam for 48 hours before being housed in individual pens, were housed on clean straw with access to clean drinking water. All calves were offered 5 L of milk replacer each morning and concentrates from D7 onward. Calves in the D7 – D96 group were given calf starter at the following rates; D7 – D14 at 300 g/d, D14 - D21 at 500 g/d, D21 – D28 at 700 g/d, 1 kg/day until weaning and *ad libitum* access thereafter. The calves in D96 cohort were weaned on D56 and had *ad libitum* access to calf starter, hay and water for the remaining trial duration. The same procedure for euthanisation and sample collection was followed at all other time points.

### Extraction and amplification

Colon digesta samples were ground down to a fine powder under constant liquid nitrogen using a chilled and sterile pestle and mortar [28,29]. The DNA was extracted from approximately 250 mg of ground frozen sample using a modified version of the repeated bead beating and column purification method as previously described [30]. All extractions included a blank negative control. DNA quality was assessed using agarose gels (0.8%) and a 1-kb DNA ladder (Bioline GmbH, Luckenwalde, Germany). The concentration of extracted DNA was quantified on the Nanodrop 1000 spectrophotometer.

### 16S rRNA gene amplicon library preparation and sequencing

Using 12.5 ng of extracted rumen microbial DNA, amplicon libraries (n = 42) were created through two rounds of PCR amplification following the guidelines set in the Illumina MiSeq 16S Sample Preparation Guide, with minor adjustments to the cycle length [31]. Sequence run performance and library preparation was assessed by the generation of six amplicon libraries. Three amplicon libraries (n = 3) were generated using the three different aliquots ZymoBIOMICS*™* Microbial Community DS (Zymo Research Corp., Irvine, CA, United States). The V4 hypervariable region of the 16S rRNA gene was amplified in the first PCR using the 515F/806R primers [32], designed with Nextera overhang adapters and 2x KAPA Hifi HotStart ReadyMix DNA polymerase (Roche Diagnostics, West Sussex, United Kingdom). PCR reactions were conducted using the following conditions: initial denaturation step at 95 °C for 3 minutes, followed by 20 cycles of denaturation at 95 °C for 30 seconds, annealing at 55 °C for 30 seconds, extension at 72 °C for 30 seconds, and a final extension at 72 °C for 5 minutes.

The QIAquick PCR Purification Kit (Qiagen, Manchester, United Kingdom) was used to purify the amplicons, and each set of reactions included a negative control prepared using molecular water. Following purification, a second PCR was conducted to attach dual indices and Illumina sequencing adapters using the Nextera XT indexing kit (Illumina, San Diego, CA, United States) under the following conditions; initial denaturation at 95 °C for 3 minutes, followed by 8 cycles of denaturation at 95 °C for 30 seconds, annealing at 55 °C for 30 seconds, extension at 72 °C for 30 seconds, and a final extension at 72 °C for 5 minutes. A final PCR purification step was carried out as described above using the QIAquick PCR Purification Kit (Qiagen, Manchester, United Kingdom). Amplicon integrity was assessed in a 2% agarose gel. Amplicons were pooled into three separate batches for sequencing. All libraries were combined at equal concentrations and purified to remove adapter primers using the Qiagen Gel Extraction Kit (Qiagen, Manchester, United Kingdom) with further purification to eliminate potential agarose residues using the QIAquick PCR purification kit (Qiagen, Manchester, United Kingdom).

Pooled sample purity and quantity was assessed using the Nanodrop 1000, and validated using a Qubit fluorometer and using the KAPA SYBR FAST universal kit with the Illumina Primer Premix (Roche Diagnostics, West Sussex, United Kingdom). The pooled libraries were subsequently diluted and denatured as per the Illumina MiSeq *16S Sample Preparation Guide*. Sequencing was carried out on an Illumina MiSeq platform using the 500-cycle version 2 MiSeq reagent kit (Illumina, San Diego, CA, United States). Technical replicates and controls were included to evaluate the robustness of the methodology in a low-biomass context and to inform optimisation of future protocols.

### Bioinformatics analysis

Chimeric sequences were identified and removed using the DADA2 pipeline. On average, 0.69 ± 0.95% of merged reads were identified as chimeric, with removal rates ranging from <0.001% to 3.88%, and no samples had removal rates greater than 10%. While this is within expectation for low-biomass samples, it may impact the representativeness of low-abundance taxa. The consistently low proportion of chimeric reads observed in this study is unlikely to be due to low-quality DNA, as all samples met the input DNA threshold prior to amplification and gel electrophoresis confirmed the integrity of the extracted DNA. This suggests that chimera formation was minimal during PCR, likely reflecting the combination of high-quality template DNA and optimised amplification conditions. We note that alternate chimera detection methods, may provide further resolution in future studies [33].

The amplicon sequence data were processed in *R* (version 4.0.2) using *DADA2* (version 1.18.0), following a published pipeline [34], with minor modifications [35]. The quality of the forward and the reverse reads were evaluated by inspecting their Q scores to ensure that the average scores were above 30 for both. To improve read quality, any low-quality reads were filtered and trimmed with primers sequences using the trimLeft function. Identical reads were identified and consolidated using the de-replication feature, followed by merging of the forward and reverse reads. An amplicon sequence variant (ASVs) table was created, and chimeric sequences were removed. Taxonomic classification was assigned to the sequence variants using the RefSeq + RDP database (NCBI RefSeq 16S rRNA database supplemented by RDP) from the *DADA2* website. Due to high sequence similarity in the 16S rRNA gene, the database cannot reliably distinguish between *Escherichia* and *Shigella* at the genus level; thus, assignments for these genera are reported as *Escherichia/Shigella* throughout, in line with established practice [36]. Taxonomic classification was performed with a bootstrapping threshold of 80, by using minBoot = 80 with the assign Taxonomy function. A *phyloseq* object was formed by integrating sample metadata, sample taxonomy, and ASVs (version 1.34.9; [37]) and analysed in RStudio (version 4.3.0). Following this only bacterial ASVs were analysed beyond the phylum level.

### Statistical analysis

The GLM procedure of SAS (version 9.4) was used to assess the effect of calf age and farm on the α-diversity of the bacterial colon digesta community. The statistical model included calf age and farm as fixed effects. Calf sex did not affect alpha diversity (P < 0.10) and was subsequently excluded from the model. F-tests using Type III sums of squares were used to determine differences among means. The PDIFF option and Tukey’s test were used to conduct pairwise comparisons among means, considering distinctions significant at P < 0.05 and tendencies at P ≥ 0.05 and < 0.10.

Prior to examining the effects of calf age and farm location on the overall bacterial community structures, the homogeneity of group dispersion was evaluated between the two groups using the *betadisper* function in the R package *vegan*; Version 2.5.7 and implemented via *microbiome* [38]; version 1.12.0. Subsequently, PERMANOVA tests were conducted based on Bray-Curtis dissimilarities with 9,999 permutations, using a significance threshold of P < 0.05, to evaluate the influence of calf age and farm location on the bacterial community structure. Pairwise PERMANOVA comparisons were conducted to evaluate differences in microbial community composition between age groups (D7, D14, D21, D28, and D96) and between farms. Data was subset by time points and filtered for ASVs with a relative RA of > 0.05% within 50% of samples using *phyloseq*. Differential abundance analysis was performed using *MaAsLin2* versions 1.14.1 [39] on non-rarefied data. Prior to analysis, ASV counts were transformed into relative abundances, followed by log transformation to stabilise variances and normalise the data for downstream modelling. Calf age and farm location were considered as fixed effects. Default parameters were used, and p-values were adjusted using the Benjamini–Hochberg false discovery rate correction (q < 0.25).

## Results

### Sequence data information

While the 515F/806R primers amplify both archaeal and bacterial communities, a low archaeal abundance was detected in all samples at each time points, and therefore, archaea were excluded from all further analysis.

One sample, originating from a D14 calf, from Replicate 2 on Farm 1, was excluded from comparative statistical analyses due to its outlier profile, with over 22% of the total bacterial community comprised of *Escherichia coli* (compared to a mean of ~1% for the remaining D14 calves). Such high *E. coli* abundance is commonly associated with scouring or diarrhoeic calves, which are not representative of the healthy cohort. Its inclusion would have skewed exploratory differential abundance comparisons.

Colon samples obtained from calves euthanised at birth (D0) had an extremely low sequencing count, with a mean read count per sample of 71, ranging from 6 to 307, after quality filtering, merging, and removal of chimeric sequences. As a result, samples collected from birth were excluded from differential abundance analysis. Samples collected from D7 onward had an average of 74,307 ± 16,378 reads per sample. Based on a plateauing of the generated rarefication curve (S1 Fig), sequencing depth was deemed sufficient for preliminary compositional assessment. Mock community controls for each extraction sample batch were all highly correlated with *r*_*s*_ values ranging from 0.95 to 0.97 (P < 0.01).

### α-Diversity

**Fig 1:**
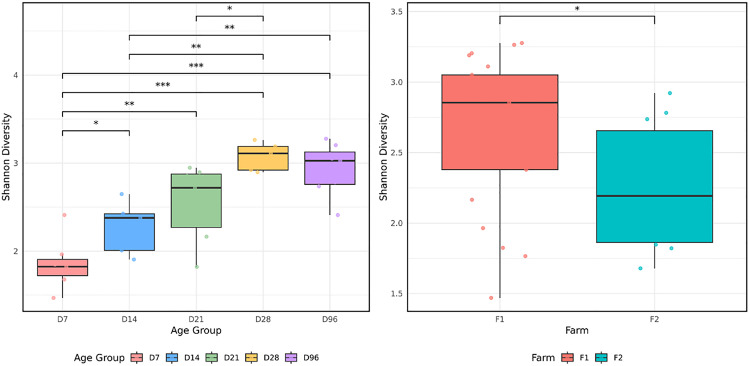
Alpha diversity metrics during early life across age (A) and both farms (B), for bacterial communities of the colon digesta content. *denotes statistically significant differences.

The bacterial α-diversity analysis was assessed using the Shannon Index (Fig 1, Table 1). Trends in alpha diversity were observed across the various time points (D7-D96), with both calf age (P < 0.01) and farm location (P < 0.01) showing the effects on community diversity. The colon digesta D14 samples tended to contain a more diverse bacteriome than the D7 samples (P = 0.07). No difference in the bacterial community diversity was detected between samples obtained at D14 and D21 (P = 0.12) or between D21 and D28 (P = 0.56). However, samples collected at D96 exhibited notably higher alpha diversity compared to earlier timepoints, particularly D7 and D14 (P < 0.05), suggesting a potential influence of dietary transition. The observations highlight the possible age and environment associated patterns in microbial diversity.

**Table 1:** Least-squares means (LSMeans) for Shannon diversity of colon digesta by age group (D7, D14, D21, D28, D96) and Tukey–Kramer–adjusted pairwise P-values from a GLM.

Group			Shannon L SMEAN		
D7			2.65		
D14			3.22		
D21			3.74		
D28			4.07		
D96			5.33		
Least squares means for effect group					
Pr > |t| for H0: LSMean(i)=LSMean(j)					
Dependent variable: Shannon					
i/j	1	2	3	4	5
1		0.07	<0.0001	<0.0001	<0.0001
2	0.07		0.12	0.01	<0.0001
3	<0.0001	0.12		0.56	<0.0001
4	<0.0001	0.01	0.56		<0.0001
5	<0.0001	<0.0001	<0.0001	<0.0001	

### β-Diversity

**Fig 2:**
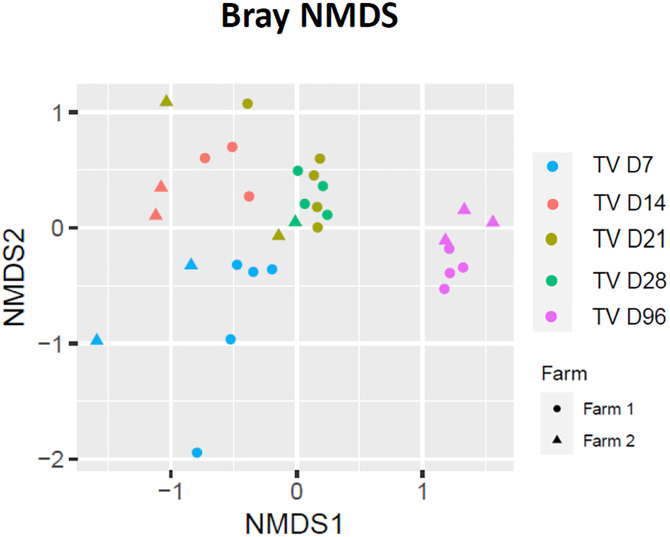
Bray- Curtis Non-dimensional multiscaling (nMDS) plot of the bacterial genus community at each of the time points sampled. The different colour dots represent the days that animals were sampled.

PERMANOVA testing based on Bray-Curtis dissimilarity matrices (Table 2), suggested that both calf age and farm were associated with differences in the composition of the colon bacterial community (P < 0.01). The bacterial community profiles were visualised using non-metric multidimensional scaling (nMDS), was used to visualise community patterns (Fig 2). The ordination plot revealed age-associated clustering, with samples from D7 and D14 appearing more distinct, while D21 and D28 clustered more closely. D96 samples formed a separate grouping at the ASV level, indicating a potential compositional shift post-weaning.

**Table 2:** Permanova results on Bray-Curtis dissimilarity matrices of rarefied ASV’s count tables for bacterial communities during early life.

Factor	^1^DF	Sum of Sq’s	R^2^	^2^F	^3^*P*-Value
Age	4	3.49	0.3	3	0
Farm	1	0.81	0.7	2.78	0
Residuals	25	7.28	0.63	–	–
Total	30	11.57	1	–	–

^1^DF - Degrees of freedom.

^2^F - Pseudo-F value, obtained by permutation.

^3^*P*-value: obtained based on 9,999 permutations (lowest *P*-value possible 0.001).

Pairwise PERMANOVA comparisons indicated temporal change in bacterial community from D7 to D14 was observed (P < 0.05), with a change in RA observed between farms (P < 0.001). Differences between farms were overserved from D14 and D21 (P < 0.01) and D21 and D28 farms (P < 0.05). This pairwise PERMANOVA comparison also demonstrated a shift in the microbial composition from D28 to D96 (P < 0.01), driven by age (P < 0.05), though there was no difference with regard to farm origin (P > 0.1). No differences in β-dispersion (homogeneity of variance) of bacterial communities were detected based on age or farm (P > 0.05).

### Taxonomic composition of the colon microbiota during early life

**Fig 3:**
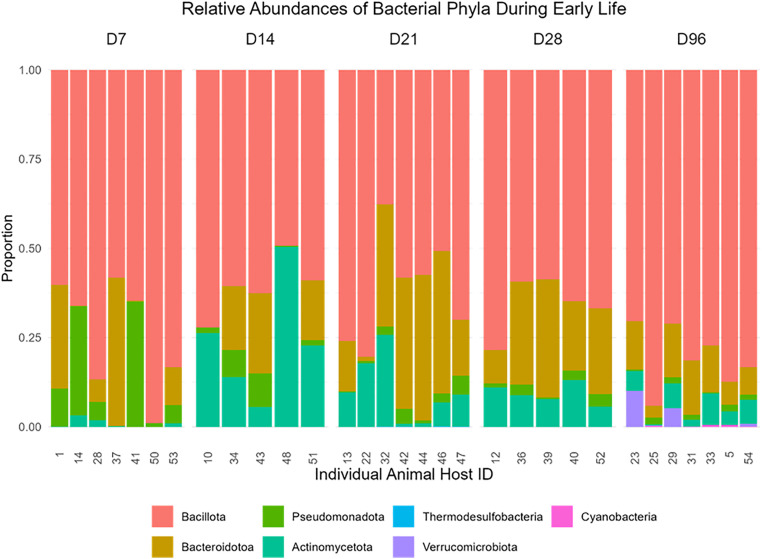
Bacterial species abundance in colon digesta during early life in the animals sampled. The values on the y-axis represent species abundances as a proportion of total bacterial phyla sequences.

**Fig 4:**
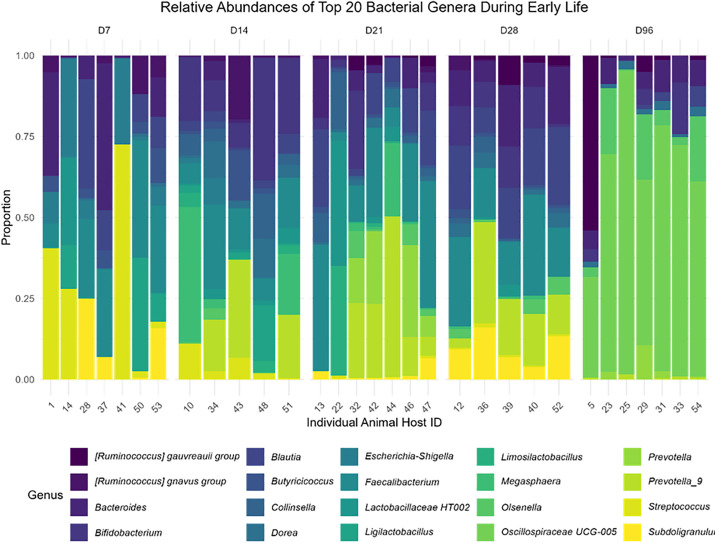
Bacterial genus abundance of the colon digesta in different time point during early life. Each bar represents an individual animal host and the x-axis shows the host ID. The y-axis depicts the proportion of the sum of the 20 most abundant bacterial genera.

Seven bacterial phyla were consistently observed across all time points sampled. The most prevalent phyla observed across all age-groups were Bacillota (formerly Firmicutes; 69.18%), Bacteroidotoa (formerly Bacteroiodetes; 16.67%), Pseudomonadota (formerly Proteobacteria; 4.65%), and Actinomycetota (formerly Actinobacteria; 8.91%). Other less abundant phyla present in the colon digesta microbiota include Thermodesulfobacteria (formerly Desulfobacteria; 0.01%), Cyanobacteria (0.53%) and Verrucomicrobiota (0.05%) (Fig 3). At the genus level *Faecalibacterium* was the most abundant taxon (10.79%) during early life, followed by *Prevotella*_9 (6.10%), unclassified *Blautia* (5.97%), *Bacteroides* (5.78%), *Oscillospiraceae* UCG-005 (5.26%), *Bifidobacterium* (5.12%) and *Streptococcus* (4.34%) (Fig 4).

### Bacterial phyla composition across all time points

Of the bacterial phyla identified, there were seven that were present in abundances of more than 5% at any one time point. Four out of the seven phyla, by D7, exhibited abundances exceeding 5%. Bacillota appeared to be the predominant phylum in the colon digesta composition across all time points, constituting an average of 84% of the content in all samples. The abundance of Bacillota remained relatively consistent, ranging from 81% to 61% from D7 to D96. In addition to Bacillota, Actinomycetota (1%), Bacteroidota (13%) and Pseudomonadota (13%) collectively constitute the remaining phyla at D7. Bacillota was established as the most dominant phyla at D96, followed by Bacteroidota (11%) and Actinomycetota (5%). Actinomycetota varied throughout the time point’s sampled, at D7 it increased from 1% to 24% at D14 (adj.P < 0.001), with a subsequently reduction at D21 (10%, adj.P < 0.05).

The relative abundance of the remaining phyla varied across timepoints but remained below the 5% threshold for most samples. Observed fluctuation in phylum-level composition overtime proves an initial overview of microbial community structure in colon digesta samples collected during early life.

### Microbial genus composition across all time points

The predominant genera present at D7 were *[Ruminococcus]* gnavus group (3.46%), *Butyricoccus* (3.57%), *Lactobacillus* HT002 (3.73%), *Subdoligranulum* (6.14%), *Ligilactobacillus* (7.46%), *Blautia* (8.33%), *Escherichia/Shigella* (9.19%), *Bacteroides* (12.57%), *Faecalibacterium* (15.65%) and *Streptococcus* (15.81%). All other genera present at D7 comprised less than 3.5% of the total bacterial sequences obtained from the colon content samples. A reduction in the RA of *Streptococcus*, was observed from 15.81% on D7 to 0.02% on D21 (D7 to D21, adjusted P < 0.001), and to less than 0.01% on D96 (D7 to D96, adjusted P < 0.01). *Faecalibacterium* showed a reduction in RA from 15.65% on D7 to less than 0.1% on D96 (adj.P < 0.001). *Escherichia/Shigella* also exhibited differenced in RA; at D7 the RA was 9.19% was different compared to D14 (1.73%) and < 0.01% for D21, D28, D96 (D7 vs. D14 adj.P = 0.05; D7 vs. D21 adj.P < 0.001; D7 vs. D28 adj.P < 0.001; D7 vs. D96 adj.P < 0.001).

[*Ruminococcus] Gnavus group* (3.41%), *Acidaminococcus* (3.64%), *Streptococcus* (4.00%), *Ligilactobacillus* (4.17%), *Collinsella* (5.23%), *Dorea* (5.27%), *Prevotella_9* (10.28%), *Faecalibacterium* (11.3%), *Megasphaera* (11.87%) and *Bifidobacteria* (17.84%) were the most dominant genera found at D14. There were variations in RA of *Bifidobacterium* at D14, compared to D7 (adj.P < 0.001), D21 (adj.P < 0.05) and D96 (adj.P < 0.05). The RA of *Bifidobacterium* increased from < 0.01% at D7 to 17.85% by D14. *Bifidobacterium* further fluctuated in abundance for the remainder of the time points; D21 (4.12%), D28 (5.95%), and D96 (1.56%). Differences were observed in the RA of *Megasphaera* at D7 (adj.P < 0.01) and D96 (adj.P < 0.05). *Prevatella_9* and *Dorea* both exhibited a change in RA from D7 to D14 (both adj.P < 0.001).

The most abundant genus by D21 include *Megasphaera* (3.06%), *Collinsellsa* (3.97%), *Bifidobacterium* (4.12%), *Bacteroides* (4.40%), *Limosilactobacillus* (4.63%), *Lactobacillus HT002* (5.74%), *Prevotella* (7.28%), *Blautia* (8.05%), *Prevotella_9* (12.29%) and *Faecalibacterium* (15.39%). There was a difference in the RA of *Faecalibacterium* between D21 and D96 from 15.39% to < 0.01% (adj.P < 0.001). *Bifidobacterium* showed differences in RA between D21 and at D7 (adj.P < 0.01) and D96 (adj.P < 0.1). *Blautia* exhibited a difference in RA between D21 and D96 (adj.P < 0.05). Additionally, *Lactobacillus HT002* experienced a reduction in RA (adj.P < 0.05), from 5.74% to < 0.01%. Differences in RA of *Faecalibacterium* and *Blautia* observed between D28 and D96 (adj.P < 0.05 and adj.P = 0.01 respectively). *Subdoligranulum* displayed a change in RA from D28 to D14 (adj.P < 0.001) and D28 vs. D96 (adj.P < 0.001). There was a change observed in the RA of *Bifidobacteria* between D28 and D7 (adj.P < 0.001) as well as D28 and D96 (adj.P < 0.001).

The most abundant genera identified at D96 included *Bacteroides* (2.39%), *Christensenellaceae* R-7 group (2.47%), *Acetitomaculum* (2.79%), *Rikenellaceae* (3.09%), *Olsenella* (3.09%), *[Eubacterium] coprostanoligenes group* unclassified (3.22%), [*Ruminococcus] gauvreauii* group unclassified (4.58%), *Oscillospiraceae UCG-010* (4.62%), *Lachnospiraceae* unclassified (10.53%) and *Oscillospiracese UCG-005* (23.1%). The RA of *Oscillospiracese UCG-005, Lachnospiraceae* unclassified, *Oscillospiraceae UCG-010,* and *[Eubacterium] coprostanoligenes group* unclassified differed at D96 compared to earlier time points. Additionally, *[Ruminococcus] gauvreauii* group unclassified differed between D96 and both D7 (adj.P < 0.001) and D14 (adj.P < 0.1).

### Farm specific genus composition

Bacterial genus comparisons of bacterial genus RA between F1 and F2 suggested differences at multiple time points. At D7, D28, and D96, the RA of the following genera appeared higher in F1 compared to F2: *unclassified Prevotellaceae*, *Prevotella_9*, *Dialister*, *Acidaminococcus*, *unclassified Atopobiaceae*, and *Prevotella* (all with adjusted p-values < 0.05). Conversely, the RA of the *[Eubacterium] nodatum group* was lower in F2 than in F1 at D7 (adj.P < 0.1), with this pattern continuing at D28 and D96 (adj. P < 0.1 for both time points). *Unclassified Gastranaerophilales* had a higher abundance in F2 compared to F1 across all time points sampled (adj.P < 0.05). *Ligilactobacillus* showed a higher RA in F2 compared to F1 at D7, D28 and D96 (adj.P < 0.05, adj.P < 0.05 and adj.P < 0.005 respectively). Unclassified *[Eubacterium] coprostanoligenes* group, *Rikenellaceae RC9* gut group and *Alloprevotella* showed reduced abundance in F2 when compared to F1 (adj.P < 0.05, adj.P < 0.05 and adj.P < 0.05, respectively). *Collinsella* and *Anaerovibrio* appeared to have higher RA in F2 than in F1 (adj.P < 0.05 for both).

## Discussion

Understanding the temporal establishment of the lower GIT microbial composition in calves is essential for developing strategies that enhance host performance. The colon microbiome plays a pivotal role in digestion, immune function, and overall health, directly affecting calf welfare and development. The balance of microbial populations influences nutrient absorption and the fermentation of milk prior to the rumen becoming fully functional [40].

This study, constrained by sample availability, utilised direct colon digesta to explore temporal and farm-specific shifts in the colon microbiota of Angus beef calves from birth to post-weaning, indicating increased microbial diversity and a distinct age-related change in taxonomic composition. Unlike most neonatal microbiome studies that rely on faecal or rumen samples [41,42], this work focuses on destructively samples colon digesta, providing a more direct presentation of the lower GIT microbiota. The sampling calves across six early-life stages, from birth to post-weaning, enabled high resolution perspective of colon microbial development.

The relative abundance of key bacterial taxa, including *Faecalibacterium*, *Prevotella*_9, and *Bifidobacterium*, varied significantly across time points, while farm location influenced microbial community structure, particularly at D7, D28, and D96. These findings are consistent with patterns seen in rumen microbial dynamics in the same animal cohort [25], reinforcing the concept of age-driven microbial development in the ruminant GIT. Both of the studies underscore age-driven microbial succession, with clear shifts in diversity and composition over time, and pronounced effects of weaning on microbial communities. However, distinct functional roles were observed between ecosystems with the rumen microbiota is dominated by fibrolytic taxa supporting plant fibre degradation, while the colon microbiota primarily comprised taxa involved in short-chain fatty acid (SCFA) production, supporting to hindgut fermentation, suggesting distinct but complementary microbial functions across GIT regions.

Bacterial α-diversity in the colon, as measured by the Shannon diversity index, appeared to increase progressively over time from D7 to D96, suggesting a trend toward microbial maturation. This observation is broadly consistent with previous studies, which reported that the colon microbiome reached a comparable level of homogeneity by four weeks of age compared to the rumen microbiota of calves at the same age, highlighting differences in microbial stabilisation [43]. β-diversity analyses, including nMDS, illustrated temporal clustering and pronounced shifts in community structure as calves matured. Similar to prior findings, Bacillota, Bacteroidota, and Pseudomonadota were also identified as the most prevalent phyla in the hindgut of calves from birth to seven weeks, with Bacillota being the dominant phylum [40]. One explanatory observation of particular interest is the potential plateau in microbiota composition between D14 and D21, which may represent a critical window for future microbiome modulation interventions.

Compared to previous studies on the rumen, the colon microbiota in this study appeared more dynamic, with greater shifts in α-diversity over time. This may reflect the colon’s responsiveness to changing nutrient inputs during transition from a milk-based to solid feed diet. The colon, therefore play a critical role in adapting to shifting nutrient inputs during the dietary transition from milk to solid feed, particularly as the rumen becomes functional. The gradual increase in undigested dietary components reaching the colon during this developmental period could contribute to the observed variability in microbial diversity and composition.

In this study, bacterial α-diversity in the colon, as measured by the Shannon diversity index, showed a proportional increase from D7 to D96, suggesting a trend toward microbial maturation with age. While age- and farm-related effects on the rumen microbiota have been reported previously, the structural changes observed were less pronounced than those in the colon [25]. These preliminary observations highlight the colon’s heightened variability and dynamic shifts during the critical developmental window from birth to weaning. The colon’s dynamic nature underscores its pivotal role in early-life health and development with implications for nutrient absorption and possible interactions with host immune development, although direct evidence of immunological measurements were not assessed in this study.

The observed progression of the calf colon microbiota from D7 to D96 reflects age-associated trends in both diversity and composition. While this is consistent with findings from previous studies [40,43], our study provides a unique perspective by directly sampling colon digesta across six early developmental time points. At D0, the bacterial diversity in the colon was notably low, consistent with a limited perinatal period, and increased with calf-age, potentially supporting enhanced microbial resilience and colonisation resistance over time.

Statistical analyses, including the Shannon diversity index and β-diversity measures, suggest that age was a key factor shaping the colon bacterial diversity and community composition, with increasing in complexity observed from D7 onwards. A period of apparent stabilisation of the colon microbiome between D14 and D21 may represent a transitional phase in microbiota development, mirroring trends previously reported in rumen microbial succession within the same cohort [25]. The possibility of colon microbiota stabilisation at three weeks of age, indicates that interventions before D21 could represent an optimal window to influence microbial composition, enhancing gut health and nutrient utilisation.

In addition to changes in bacterial diversity, age-associated trends in bacterial genus across the sampled time points were observed. *Escherichia/Shigella* showed a notable decrease in relative abundance from D7 (9.19%) to D14 (1.7%), illustrating the dynamic nature of early microbial colonisation of the colon digesta. This reduction may be driven by several factors, including the maturation of the host immune system, competition with other microbes, and dietary transitions. In the early neonatal period, *Escherichia/Shigella,* may transiently dominate due to the immature immune environment and the availability of simple sugars such as lactose, which it can efficiently metabolise. As the colon microbiota matures and diversifies, opportunistic taxa such as, *Escherichia/Shigella* may be gradually outcompeted by commensals better adapted to the maturing gut environment.

This apparent decline in, *Escherichia/Shigella* could also coincide with the re-establishment of the SCFA synthesis and the recovery of key metabolic pathways, including butyrate production, which are essential for maintaining gut health [44]. *Escherichia/Shigella.* has been associated with increased incidence of diarrhoeal disease in calves and is often found in higher abundance in diseased animals [28].

Beyond its pathogenic associations, *Escherichia/Shigella* may play a key role in early microbial ecosystem structuring. Previous work has shown that *Escherichia/Shigella* can act as a central node in shaping microbial communities, influencing both competitive and cooperative interactions [12]. In this study, the early relative abundance of *Escherichia/Shigella* likely reflects environmental exposure, and host-related factors, and may also shape the trajectory of microbial succession across calves. Although we did not directly test ecological interactions or causality, we hypothesize that *Escherichia/Shigella* plays a transient yet influential role in early colon community assembly. Future work such as time-resolved co-occurrence/network analyses and functional profiling (shotgun metagenomics or metatranscriptomics)—could clarify mechanisms and the longer-term implications for gut development and host health.

One calf in this study exhibited an exceptionally high RA of *Escherichia/Shigella*, markedly diverging from the rest of the cohort. This sample was excluded from comparative statistical analyses due to its outlier profile; however, its microbial composition remains noteworthy. Similar profiles have been described in diarrhoeic calves and may reflect a perturbed colonisation process or microbial imbalance [11,12]. Although speculative, such cases could represent disruptions in community assembly and may offer insight into how opportunistic taxa shape microbiome structure. Longitudinal studies integrating metagenomics or microbial interaction modelling would be beneficial to clarify these ecological dynamics.

In early life, the colon microbiota appears to be dominated by genera such as *Streptococcus* and *Bifidobacterium,* suggesting an influence of neonatal dietary inputs on microbial composition. *Streptococcus*, accounted for 15.8% of the microbial community at D7, it is thought to play a key role in creating an anaerobic environment by utilising oxygen [18,19]. Although *Streptococcus* and *Bifidobacterium* are often linked to immune modulation, the absence of direct immunological data in this study limits interpretation of potential functional host-microbiota interactions. Future studies incorporating mucosal gene expression or immunological markers will be necessary to explore these relationships more directly. This early dominance is likely driven by the availability of simple sugars, such as lactose, in the neonatal diet and reduced competition in the immature gut ecosystem. Oxygen depletion mediated by *Streptococcus* may further facilitate the establishment of obligate anaerobes that are important for the development of a stable and mature microbiota.

Transitioning from a milk-based diet to solid feed during weaning was associated with changes in the microbial composition of the colon. Genera such as *Streptococcus* and *Bifidobacterium*, were more prominent in early life, producing lactic acid and digesting milk-derived nutrients, which highlights the potential role of neonatal diet in shaping microbial populations important for calf growth and health [45]. The metabolic activity of facultative anaerobes, through oxygen utilisation, may support the establishment favourable conditions for obligate anaerobes, promoting the establishment of a mature microbial community.

*Bifidobacterium* showed a temporal pattern, peaking at D14 (17.84%) before declining by D21 (5.95%), a pattern that is likely influenced by dietary shifts and host development. As a lactic acid producer, *Bifidobacterium* has been associated with neonatal health and immunity [26], and may contribute to reducing opportunistic pathogens [46]. Evidence from metagenomic studies in humans have highlighted *Bifidobacterium* as a potential microbial biomarker for autoimmune diseases and as a modulator of immunotherapy efficacy in cancer treatment [47]. The observed decline from D14 onward is likely multifactorial, and consistent with foregut maturation as starter consumption increases, the rumen community shifts toward obligate anaerobes and fibrolytic taxa and SCFA output rises (notably butyrate), lowering rumen pH and accelerating papillary growth [48–51]. This re-partitions fermentation to the rumen and changes the amount and chemistry of substrates that reach the colon, plausibly driving the colon patterns we observe [48,50]. In addition, evidence that early-life interventions can “program” the rumen microbiome and fermentation supports this developmental linkage [52]. Moreover, microbiome stability increases with age, so both rumen and downstream colon communities tend to show reduced variability as calves transition to solid feed [42]. Previous research suggests that such reductions, may be part of a coordinated process during early developmental [11]. These observations emphasise the effects of age and diet on the microbial community assembly and highlight the importance of longitudinal studies to disentangle these factors. Larger studies will be needed to confirm the role of B*ifidobacterium* in the establishment of resilient and function calf microbiome.

By D96, the colon bacterial community appeared to be increasingly dominated by fibre- and starch-degrading taxa, such as *Oscillospiraceae UCG-005* (23.1%) and unclassified *Lachnospiraceae* (10.53%) suggesting the adaptation to a fibrous, solid-feed diet. This transition was accompanied by a decline in *Faecalibacterium* and *Streptococcus*. *Faecalibacterium*, a butyrate-producing genus, decreased sharply from D7 (15.65%) to D96 (<0.01%), likely as a result of competition from microbes better attuned to the evolving gut environment. Beyond its metabolic role, *Faecalibacterium* has been identified as a significant driver of microbial assembly, particularly in diarrheic calves [11], and contributing to shaping early microbial community structure. Despite its decline, *Faecalibacterium* remains functionally relevant, having been associated with reduced inflammation, improved weight gain, and lower incidence of diarrhoea.

The progressive increase in bacterial diversity from D7 to D96 is likely driven by GIT maturation, including enhanced mucosal secretions and a developing immune system. Gut-associated lymphoid tissues (GALT) and the mucosal barrier play critical roles in maintaining gut integrity and supporting beneficial microbial colonisation [53,54]. Early colonisation by beneficial microbes plays a pivotal role in supporting immune system maturation and robust gut barrier function, critical for maintaining intestinal health and resilience.

A strength of this study is the use of a commercially relevant beef calf cohort across two different farms adds environmental which introduces environmental and management variability often missing from controlled studies. Although the present study did not directly assess transport effects on the gut microbiome, previous research has demonstrated that long-distance transportation can alter the composition of microbial communities in cattle, particularly within the respiratory tract [55] although no differences in faecal microbial alpha or beta diversity was detected in a study where young calves were transport up to 16hrs [56]. In this study, transport duration lasted approximately two hours and was conducted under controlled and low-stress conditions. Considering that more pronounced effects have been reported following longer journeys, we consider it unlikely that such short-term transportation had a major or lasting impact on the maternal or neonatal gut microbiome in our experimental setting.

The collection of faecal samples, while being more convenient and less invasive, has typically been used as a representative of the GIT microbiome. There is a substantial amount of research available on calf faecal microbiomes in relation to health and diarrhoeal disease [57]. In a previous study, faecal and inner colonic communities shared broad taxonomic signatures but showed significant between-site differences in beta-diversity and the relative abundance of mucosa-associated genera, with the faecal samples providing coarse-level comparability but are not substitutes for inner colonic microbial profiles [58]. Less invasive approaches, such as oral swabs, albeit indirect, have shown partial alignment with rumen microbiota and can rank animals by broad community shifts and relate to feed-intake proxies, but their predictive performance is context-dependent and modest [59]. In chickens, faecal samples have been found useful for detecting a wide variety of microbial species within the GIT. However, it remains unclear which region of the GIT these microbes represent [60]. While faecal collection is undoubtedly a less-invasive method, it does not fully represent the true composition of the lower GIT, and therefore any of the derivative data should be interpreted with a caution.

While this study provides insights into the temporal and compositional dynamics of the calf colon microbiota, it is important to acknowledge several limitations. Given the limited sample size, the study may not have captured all biologically relevant differences, and some existing effects may have gone undetected. The calculated requirement for adequate statistical power was n = 7 per timepoint, with a 20% coefficient of variation and detection of a 30% difference in mean values at 80% power and α = 0.05. Two time points (D14, D28) had five animals; a retrospective calculation (solving for the standardized effect size that yields 80% power at n = 7) implies approximately 62% power at n = 5. This reduction in sample size likely limited the ability to detect some biologically relevant effects and means the results should be interpreted with appropriate caution and as indicative rather than definitive. Although we employed a validated, robust extraction protocol, all DNA extraction methods may underrepresent low-abundance bacterial taxa- particularly in low biomass samples such as the neonatal colon digesta. Mock community controls demonstrated high extraction efficiency and sequencing and sequencing depth was sufficient; it remains possible that rare or low-abundance taxa were not detected. The 16S rRNA gene sequencing provides valuable information on microbial community composition, it captures relative rather than absolute abundances and is therefore subject to compositional constraints. Apparent changes in the relative abundance of individual taxa may reflect fluctuations in the overall community structure rather than true shifts in microbial load. Consequently, caution is warranted when interpreting taxonomic changes as indicators of biological function.

Nevertheless, the key bacterial genera identified, such as including *Faecalibacterium*, *Bifidobacterium*, and *Streptococcus* have been widely associated with critical metabolic and immunological roles in the gut. For example, *Faecalibacterium* is a known butyrate producer with anti-inflammatory properties, *Bifidobacterium* supports mucosal health and immune modulation through SCFA production, and early colonisation by *Streptococcus* contributes to the creation of an anaerobic environment essential for microbiota development. The absence of functional or immunological profiling in this study limits the ability to confirm these host-microbial interactions.

Similarly, while *in vitro* culturing could provide further functional insights, its application to low-biomass samples like neonatal colon digesta is technically challenging and was not included in this preliminary study. Future studies would be strengthened by integrating quantitative methods, such as spike-in controls or qPCR, in combination with metagenomic, transcriptomic, or immunological analyses to more robustly characterise microbiota-host interactions.

Despite these limitations, the current study offers a detailed, longitudinal characterisation of colon microbiota development in calves and lays the groundwork for future research into early-life microbial succession in the lower gastrointestinal tract.

The pronounced changes in diversity and microbial composition observed from D7 to D96 highlight the potential importance of the colon microbiota in early-life health and development. These findings suggest that age, diet, and immune system maturation are key shaping forces, and reinforce the importance of targeted interventions during the critical period from birth to weaning to promote a resilient and functionally beneficial microbiota.

## Conclusion

The neonatal GIT microbiome is highly complex and plays a pivotal role in immune development, nutrient absorption, and disease resistance. This study aimed to explore the temporal establishment of the colon microbiota in Aberdeen Angus calves from birth to post-weaning, providing insights into the age-dependent dynamics of microbial colonisation. The data suggests that the colon digesta-associated microbiota undergoes considerable changes in response to various factors such as environment and dietary changes, particularly as calves increase their consumption of calf starter from D7 through to D96 (post-weaning).

A period of relative compositional similarity prior to D21, followed by an apparent shift in microbial structure post-weaning. This suggests may indicate that during early life, the rate of compositional change slows, before weaning introduces further complexity and diversification to support the calf’s transition to solid feed. These findings reinforce the importance of understanding age-dependent microbial dynamics for optimising calf health and performance. Future studies involving larger samples sizes and the application of multi-omics techniques could provide deeper insights into the complex interactions involved in colon microbiota colonisation during early life.

When considered alongside existing knowledge of rumen microbial development, the data toward distinct variability between GIT compartments. Such differences underscore the potential value of compartment-specific research interventions. This distinction suggests that targeted interventions should consider the specific developmental needs of different GIT compartments to optimise the overall health in calves. In summary, this study offers a foundation for future research aimed at developing stage-specific, evidence-based interventions to support a beneficial and functional colon microbiome from neonatal development through to maturity.

## Supporting information

S1 Fig Rarefication curve illustrating sequencing depth.
(JPG)

## References

[1] Arshad MA, Hassan F-U, Rehman MS, Huws SA, Cheng Y, Din AU. Gut microbiome colonization and development in neonatal ruminants: Strategies, prospects, and opportunities. Anim Nutr. 2021;7(3):883–95. doi: 10.1016/j.aninu.2021.03.004 34632119 PMC8484983

[2] Myer PR, Wells JE, Smith TPL, Kuehn LA, Freetly HC. Microbial community profiles of the colon from steers differing in feed efficiency. Springerplus. 2015;4:454. doi: 10.1186/s40064-015-1201-6 26322260 PMC4549364

[3] Xu Q, Qiao Q, Gao Y, Hou J, Hu M, Du Y. Gut Microbiota and Their Role in Health and Metabolic Disease of Dairy Cow. Front Nutr. 2021;8:701511. 34422882 10.3389/fnut.2021.701511 PMC8371392

[4] Zhang Y, Choi SH, Nogoy KM, Liang S. Review: The development of the gastrointestinal tract microbiota and intervention in neonatal ruminants. Animal. 2021;15(8):100316. doi: 10.1016/j.animal.2021.100316 34293582

[5] Meade KG, O’Farrelly C. β-Defensins: Farming the Microbiome for Homeostasis and Health. Frontiers in Immunology. 2019;9. 30761155 10.3389/fimmu.2018.03072 PMC6362941

[6] Jin C, Wu S, Liang Z, Zhang J, Lei X, Bai H, et al. Multi-omics reveal mechanisms of high enteral starch diet mediated colonic dysbiosis via microbiome-host interactions in young ruminant. Microbiome. 2024;12(1):38. doi: 10.1186/s40168-024-01760-w 38395946 PMC10893732

[7] Godden SM, Lombard JE, Woolums AR. Colostrum Management for Dairy Calves. Vet Clin North Am Food Anim Pract. 2019;35(3):535–56. doi: 10.1016/j.cvfa.2019.07.005 31590901 PMC7125574

[8] Lichtmannsperger K, Hartsleben C, Spöcker M, Hechenberger N, Tichy A, Wittek T. Factors Associated with Colostrum Quality, the Failure of Transfer of Passive Immunity, and the Impact on Calf Health in the First Three Weeks of Life. Animals (Basel). 2023;13(11):1740. doi: 10.3390/ani13111740 37889665 PMC10251921

[9] Immler M, Büttner K, Gärtner T, Wehrend A, Donat K. Maternal Impact on Serum Immunoglobulin and Total Protein Concentration in Dairy Calves. Animals (Basel). 2022;12(6):755. doi: 10.3390/ani12060755 35327151 PMC8944455

[10] Raboisson D, Trillat P, Cahuzac C. Failure of Passive Immune Transfer in Calves: A Meta-Analysis on the Consequences and Assessment of the Economic Impact. PLoS One. 2016;11(3):e0150452. doi: 10.1371/journal.pone.0150452 26986832 PMC4795751

[11] Frazier AN, Ferree L, Belk AD, Al-Lakhen K, Cramer MC, Metcalf JL. Stochasticity Highlights the Development of Both the Gastrointestinal and Upper-Respiratory-Tract Microbiomes of Neonatal Dairy Calves in Early Life. Animals (Basel). 2025;15(3):361. doi: 10.3390/ani15030361 39943131 PMC11816138

[12] Pan Z, Ma T, Steele M, Guan LL. Varied microbial community assembly and specialization patterns driven by early life microbiome perturbation and modulation in young ruminants. ISME Commun. 2024;4(1):ycae044. doi: 10.1093/ismeco/ycae044 38650709 PMC11033733

[13] Furman O, Shenhav L, Sasson G, Kokou F, Honig H, Jacoby S, et al. Stochasticity constrained by deterministic effects of diet and age drive rumen microbiome assembly dynamics. Nat Commun. 2020;11(1):1904. doi: 10.1038/s41467-020-15652-8 32312972 PMC7170844

[14] Husso A, Lietaer L, Pessa-Morikawa T, Grönthal T, Govaere J, Van Soom A. The Composition of the Microbiota in the Full-Term Fetal Gut and Amniotic Fluid: A Bovine Cesarean Section Study. Front Microbiol. 2021;12. 10.3389/fmicb.2021.626421 PMC8119756 33995290

[15] Banchi P, Colitti B, Opsomer G, Rota A, Van Soom A. The dogma of the sterile uterus revisited: does microbial seeding occur during fetal life in humans and animals?. Reproduction. 2023;167(1):e230078. doi: 10.1530/REP-23-0078 37903182 PMC10762539

[16] Amat S, Holman DB, Schmidt K, McCarthy KL, Dorsam ST, Ward AK, et al. Characterization of the Microbiota Associated With 12-Week-Old Bovine Fetuses Exposed to Divergent in utero Nutrition. Front Microbiol. 2022;12:771832. doi: 10.3389/fmicb.2021.771832 35126326 PMC8811194

[17] Kennedy KM, de Goffau MC, Perez-Muñoz ME, Arrieta M-C, Bäckhed F, Bork P, et al. Questioning the fetal microbiome illustrates pitfalls of low-biomass microbial studies. Nature. 2023;613(7945):639–49. doi: 10.1038/s41586-022-05546-8 36697862 PMC11333990

[18] Malmuthuge N, Griebel PJ, Guan LL. The gut microbiome and its potential role in the development and function of newborn calf gastrointestinal tract. Front Vet Sci. 2015;2:36. doi: 10.3389/fvets.2015.00036 26664965 PMC4672224

[19] Lyons T, Jahns H, Brady J, O’Hara E, Waters SM, Kenny D, et al. Integrated analyses of the microbiological, immunological and ontological transitions in the calf ileum during early life. Sci Rep. 2020;10(1):21264. doi: 10.1038/s41598-020-77907-0 33277514 PMC7718239

[20] Amin N, Seifert J. Dynamic progression of the calf’s microbiome and its influence on host health. Comput Struct Biotechnol J. 2021;19:989–1001. doi: 10.1016/j.csbj.2021.01.035 33613865 PMC7868804

[21] Celi P, Cowieson AJ, Fru-Nji F, Steinert RE, Kluenter A-M, Verlhac V. Gastrointestinal functionality in animal nutrition and health: New opportunities for sustainable animal production. Animal Feed Sci Technol. 2017;234:88–100. doi: 10.1016/j.anifeedsci.2017.09.012

[22] Heller MC, Chigerwe M. Diagnosis and Treatment of Infectious Enteritis in Neonatal and Juvenile Ruminants. Vet Clin North Am Food Anim Pract. 2018;34(1):101–17. doi: 10.1016/j.cvfa.2017.08.001 29275032 PMC7125638

[23] Todd CG, McGee M, Tiernan K, Crosson P, O’Riordan E, McClure J, et al. An observational study on passive immunity in Irish suckler beef and dairy calves: Tests for failure of passive transfer of immunity and associations with health and performance. Prev Vet Med. 2018;159:182–95. doi: 10.1016/j.prevetmed.2018.07.014 30314781

[24] Abuelo A, Cullens F, Brester JL. Effect of preweaning disease on the reproductive performance and first-lactation milk production of heifers in a large dairy herd. J Dairy Sci. 2021;104(6):7008–17. doi: 10.3168/jds.2020-19791 33685674

[25] O’Hara E, Kenny DA, McGovern E, Byrne CJ, McCabe MS, Guan LL, et al. Investigating temporal microbial dynamics in the rumen of beef calves raised on two farms during early life. FEMS Microbiol Ecol. 2020;96(2):fiz203. doi: 10.1093/femsec/fiz203 31917419

[26] Raabis S, Li W, Cersosimo L. Effects and immune responses of probiotic treatment in ruminants. Vet Immunol Immunopathol. 2019;208:58–66. doi: 10.1016/j.vetimm.2018.12.006 30712793 PMC6526955

[27] Surlis C, McNamara K, O’Hara E, Waters S, Beltman M, Cassidy J, et al. Birth delivery method affects expression of immune genes in lung and jejunum tissue of neonatal beef calves. BMC Vet Res. 2017;13(1):391. doi: 10.1186/s12917-017-1310-2 29237479 PMC5729508

[28] Scully S, Earley B, Smith PE, McAloon C, Waters SM. Health-associated changes of the fecal microbiota in dairy heifer calves during the pre-weaning period. Front Microbiol. 2024;15. 10.3389/fmicb.2024.1359611 PMC11082272 38737409

[29] Smith PE, Waters SM, Kenny DA, Boland TM, Heffernan J, Kelly AK. Replacing Barley and Soybean Meal With By-products, in a Pasture Based Diet, Alters Daily Methane Output and the Rumen Microbial Community in vitro Using the Rumen Simulation Technique (RUSITEC). Front Microbiol. 2020;11:1614. doi: 10.3389/fmicb.2020.01614 32793146 PMC7387412

[30] Smith PE, Kelly AK, Kenny DA, Waters SM. Differences in the composition of the rumen microbiota of finishing beef cattle divergently ranked for residual methane emissions. Front Microbiol. 2022;13. 10.3389/fmicb.2022.855565 PMC9099143 35572638

[31] Smith PE, Enriquez-Hidalgo D, Hennessy D, McCabe MS, Kenny DA, Kelly AK, et al. Sward type alters the relative abundance of members of the rumen microbial ecosystem in dairy cows. Sci Rep. 2020;10(1):9317. doi: 10.1038/s41598-020-66028-3 32518306 PMC7283238

[32] Caporaso JG, Lauber CL, Walters WA, Berg-Lyons D, Lozupone CA, Turnbaugh PJ, et al. Global patterns of 16S rRNA diversity at a depth of millions of sequences per sample. Proc Natl Acad Sci U S A. 2011;108 Suppl 1(Suppl 1):4516–22. doi: 10.1073/pnas.1000080107 20534432 PMC3063599

[33] Ho M, Moon D, Pires-Alves M, Thornton PD, McFarlin BL, Wilson BA. Recovery of microbial community profile information hidden in chimeric sequence reads. Comput Struct Biotechnol J. 2021;19:5126–39. doi: 10.1016/j.csbj.2021.08.050 34589188 PMC8453192

[34] Callahan BJ, McMurdie PJ, Rosen MJ, Han AW, Johnson AJA, Holmes SP. DADA2: High-resolution sample inference from Illumina amplicon data. Nat Methods. 2016;13(7):581–3. doi: 10.1038/nmeth.3869 27214047 PMC4927377

[35] Smith PE, Waters SM, Gómez Expósito R, Smidt H, Carberry CA, McCabe MS. Synthetic Sequencing Standards: A Guide to Database Choice for Rumen Microbiota Amplicon Sequencing Analysis. Front Microbiol. 2020;11:606825. doi: 10.3389/fmicb.2020.606825 33363527 PMC7752867

[36] Chattaway MA, Schaefer U, Tewolde R, Dallman TJ, Jenkins C. Identification of Escherichia coli and Shigella Species from Whole-Genome Sequences. J Clin Microbiol. 2017;55(2):616–23. doi: 10.1128/JCM.01790-16 27974538 PMC5277532

[37] McMurdie PJ, Holmes S. phyloseq: an R package for reproducible interactive analysis and graphics of microbiome census data. PLoS One. 2013;8(4):e61217. doi: 10.1371/journal.pone.0061217 23630581 PMC3632530

[38] Lahti L, Shetty S. Tools for microbiome analysis in R Bioconductor; open source software for bio-informatics. http://microbiome.github.com/microbiome 2017.

[39] Mallick H, Rahnavard A, McIver LJ, Ma S, Zhang Y, Nguyen LH, et al. Multivariable association discovery in population-scale meta-omics studies. PLoS Comput Biol. 2021;17(11):e1009442. doi: 10.1371/journal.pcbi.1009442 34784344 PMC8714082

[40] Song Y, Malmuthuge N, Steele MA, Guan LL. Shift of hindgut microbiota and microbial short chain fatty acids profiles in dairy calves from birth to pre-weaning. FEMS Microbiol Ecol. 2018;94(3):10.1093/femsec/fix179. doi: 10.1093/femsec/fix179 29267960

[41] Meale SJ, Li S, Azevedo P, Derakhshani H, Plaizier JC, Khafipour E, et al. Development of ruminal and fecal microbiomes are affected by weaning but not weaning strategy in dairy calves. Front Microbiol. 2016;7. 10.3389/fmicb.2016.00582 PMC4853645 27199916

[42] Meale SJ, Li SC, Azevedo P, Derakhshani H, DeVries TJ, Plaizier JC, et al. Weaning age influences the severity of gastrointestinal microbiome shifts in dairy calves. Sci Rep. 2017;7(1):198. doi: 10.1038/s41598-017-00223-7 28298634 PMC5428063

[43] Castro JJ, Gomez A, White B, Loften JR, Drackley JK. Changes in the intestinal bacterial community, short-chain fatty acid profile, and intestinal development of preweaned Holstein calves. 2. Effects of gastrointestinal site and age. J Dairy Sci. 2016;99(12):9703–15. doi: 10.3168/jds.2016-11007 27720148

[44] Baltazar-Díaz TA, González-Hernández LA, Aldana-Ledesma JM, Peña-Rodríguez M, Vega-Magaña AN, Zepeda-Morales ASM. Escherichia/Shigella, SCFAs, and Metabolic Pathways - The Triad That Orchestrates Intestinal Dysbiosis in Patients with Decompensated Alcoholic Cirrhosis from Western Mexico. Microorganisms. 2022;10(6). 10.3390/microorganisms10061231 PMC9229093 35744749

[45] Alipour MJ, Jalanka J, Pessa-Morikawa T, Kokkonen T, Satokari R, Hynönen U, et al. The composition of the perinatal intestinal microbiota in cattle. Sci Rep. 2018;8(1):10437. doi: 10.1038/s41598-018-28733-y 29993024 PMC6041309

[46] Martin AJM, Serebrinsky-Duek K, Riquelme E, Saa PA, Garrido D. Microbial interactions and the homeostasis of the gut microbiome: the role of Bifidobacterium. Microbiome Res Rep. 2023;2(3):17. doi: 10.20517/mrr.2023.10 38046822 PMC10688804

[47] Longhi G, van Sinderen D, Ventura M, Turroni F. Microbiota and Cancer: The Emerging Beneficial Role of Bifidobacteria in Cancer Immunotherapy. Front Microbiol. 2020;11:575072. doi: 10.3389/fmicb.2020.575072 33013813 PMC7507897

[48] Khan S, Hansen R, Scott K, Martin J, Berry S, Stevenson M, et al. G116(P) The human gut is probably sterile at birth. Arch Dis Child. 2015;100(Suppl 3):A50.3-A51. doi: 10.1136/archdischild-2015-308599.115

[49] Liu X, Liu Q, Sun S, Sun H, Wang Y, Shen X, et al. Exploring AI-2-mediated interspecies communications within rumen microbial communities. Microbiome. 2022;10(1):167. doi: 10.1186/s40168-022-01367-z 36203182 PMC9540692

[50] Diao Q, Zhang R, Fu T. Review of strategies to promote rumen development in calves. Animals (Basel). 2019;9(8):490. doi: 10.3390/ani9080490 31357433 PMC6720602

[51] Pokhrel B, Jiang H. Postnatal growth and development of the rumen: integrating physiological and molecular insights. Biology (Basel). 2024;13(4):269. doi: 10.3390/biology13040269 38666881 PMC11048093

[52] Yáñez-Ruiz DR, Abecia L, Newbold CJ. Manipulating rumen microbiome and fermentation through interventions during early life: a review. Front Microbiol. 2015;6:1133. doi: 10.3389/fmicb.2015.01133 26528276 PMC4604304

[53] Welch CB, Ryman VE, Pringle TD, Lourenco JM. Utilizing the gastrointestinal microbiota to modulate cattle health through the microbiome-gut-organ axes. Microorganisms. 2022;10(7). 10.3390/microorganisms10071391 PMC9324549 35889109

[54] Barathan M, Ng SL, Lokanathan Y, Ng MH, Law JX. The Profound Influence of Gut Microbiome and Extracellular Vesicles on Animal Health and Disease. Int J Mol Sci. 2024;25(7):4024. doi: 10.3390/ijms25074024 38612834 PMC11012031

[55] Chai J, Liu X, Usdrowski H, Deng F, Li Y, Zhao J. Geography, niches, and transportation influence bovine respiratory microbiome and health. Front Cell Infect Microbiol. 2022;12:961644. doi: 10.3389/fcimb.2022.961644 36171758 PMC9510686

[56] Vinayamohan PG, Poelstra J, Cheng T-Y, Goetz H, Renaud DL, Gomez DE, et al. Exploring the effects of transport duration on the fecal microbial communities of surplus dairy calves. J Dairy Sci. 2024;107(6):3863–84. doi: 10.3168/jds.2023-24002 38216047

[57] Tapio I, Shingfield KJ, McKain N, Bonin A, Fischer D, Bayat AR, et al. Oral samples as non-invasive proxies for assessing the composition of the rumen microbial community. PLoS One. 2016;11(3):e0151220. doi: 10.1371/journal.pone.0151220 26986467 PMC4795602

[58] Levitan O, Ma L, Giovannelli D, Burleson DB, McCaffrey P, Vala A, et al. The gut microbiome-Does stool represent right?. Heliyon. 2023;9(3):e13602. doi: 10.1016/j.heliyon.2023.e13602 37101508 PMC10123208

[59] Marcos CN, Bach A, Gutiérrez-Rivas M, González-Recio O. The oral microbiome as a proxy for feed intake in dairy cattle. J Dairy Sci. 2024;107(8):5881–96. doi: 10.3168/jds.2024-24014 38522834

[60] Yan W, Sun C, Zheng J, Wen C, Ji C, Zhang D, et al. Efficacy of fecal sampling as a gut proxy in the study of chicken gut microbiota. Front Microbiol. 2019;10:2126. doi: 10.3389/fmicb.2019.02126 31572332 PMC6753641

