# Supplementary figures and images for: Temporal establishment of the colon microbiota in Angus calves from birth to post-weaning
Source: PLoS One. 2025 Oct 28;20(10):e0334261. doi: 10.1371/journal.pone.0334261 (PMC12561963; doi:10.1371/journal.pone.0334261)

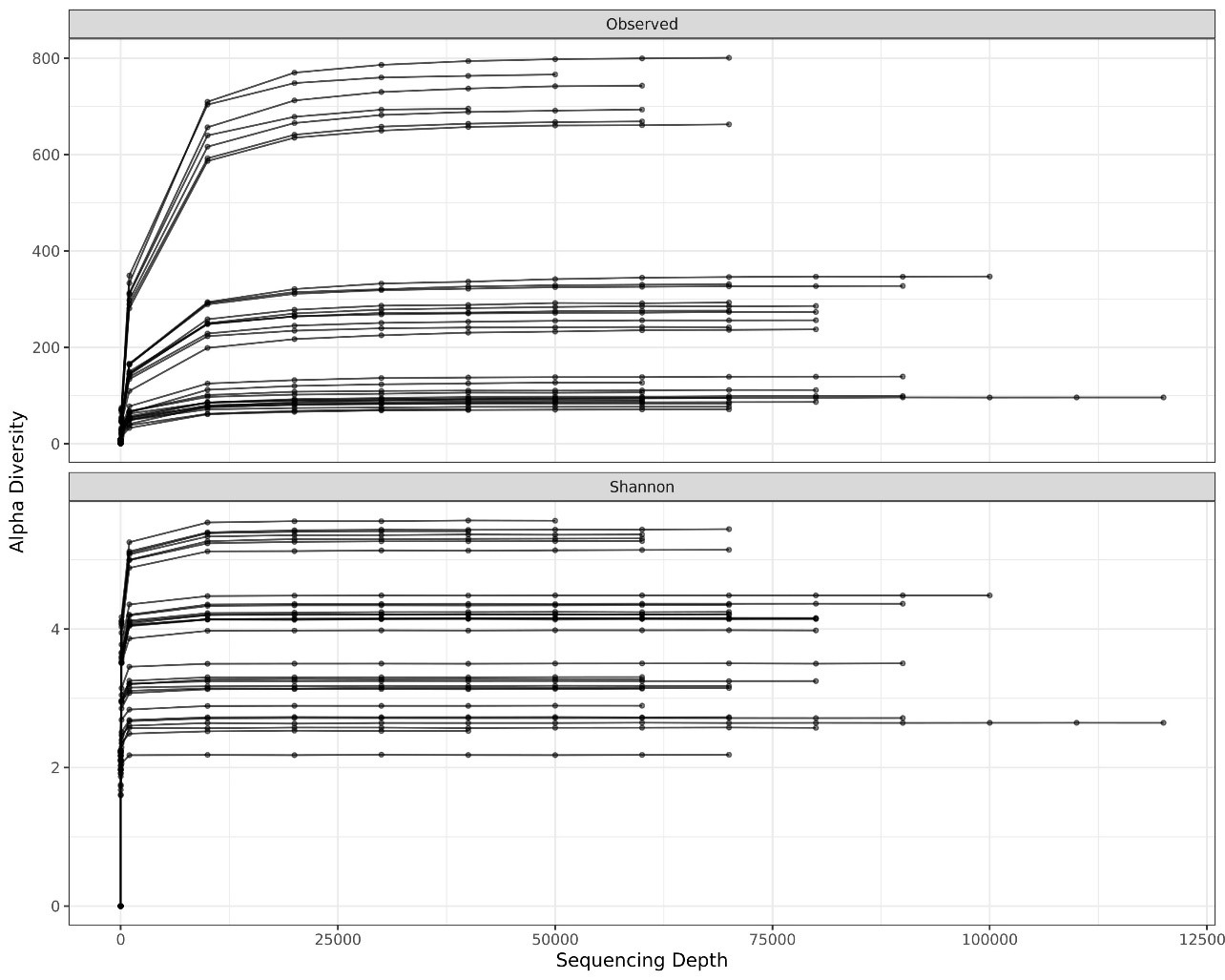

Supplement: S1 Fig — Rarefication curve illustrating sequencing depth. (JPG) [file pone.0334261.s001.jpg]
